# Retaining MKP1 expression and attenuating JNK-mediated apoptosis by RIP1 for cisplatin resistance through miR-940 inhibition

**DOI:** 10.18632/oncotarget.1798

**Published:** 2014-02-25

**Authors:** Qiong Wang, Shaoqing Shi, Weiyang He, Mabel T. Padilla, Lin Zhang, Xia Wang, Bin Zhang, Yong Lin

**Affiliations:** ^1^ Laboratory of Molecular and Translational Medicine, Key Laboratory of Birth Defects and Related Diseases of Women and Children of Ministry of Education at Sichuan University, Department of Obstetrics and Gynecology, West China Second University Hospital, Sichuan University, Chengdu, P.R. China; ^2^ Department of Urology, The First Affiliated Hospital, Chongqing, P.R. China; ^3^ Department of Social Medicine, School of Public Health, Chongqing Medical University, Chongqing, P.R. China; ^4^ Molecular Biology and Lung Cancer Program, Lovelace Respiratory Research Institute, Albuquerque, NM, USA

**Keywords:** RIP1, MKP1, JNK, cisplatin, lung cancer, apoptosis, chemoresistance

## Abstract

The elucidation of chemoresistance mechanisms is important to improve cancer patient survival. In this report, we investigated the role and mechanism through which receptor-interacting protein 1 (RIP1), a mediator in cell survival and death signaling, participates in cancer's response to chemotherapy. In lung cancer cells, knockdown of RIP1 substantially increased cisplatin-induced apoptotic cytotoxicity, which was associated with robust JNK activation. The expression of the JNK inactivating phosphatase, MKP1, was substantially reduced in RIP1 knockdown cells. Although MKP1 protein stability was not altered by RIP1 suppression, the synthesis rate of MKP1 was dramatically reduced in RIP1-suppressed cells. Furthermore, we found that the expression of miR-940 was substantially increased in RIP1 knockdown cells. Knockdown of miR-940 restored MKP1 expression and attenuated cisplatin-induced JNK activation and cytotoxicity. Importantly, ectopic expression of MKP1 effectively attenuated cisplatin-induced JNK activation and cytotoxicity. In addition, activation of the JNK upstream signaling kinase, MKK4, was also potentiated in RIP1 knockdown cells. Altogether, our results suggest that RIP1 contributes to cisplatin resistance by suppressing JNK activation that involves releasing miR-940-mediated inhibition of MKP1 and suppressing activation of MKK4. Intervention targeting the RIP1/miR-940/MKP1/JNK pathway may be used to sensitize platinum-based chemotherapy.

## INTRODUCTION

Cisplatin is a major frontline chemotherapeutic widely used to treat different cancers. Although suppression of cancer cell proliferation and angiogenesis may be involved, cisplatin directly kills cancer cells through the induction of apoptosis [1, 2]. While substantial reduction in cancer mortality and prolonged patient survival with chemotherapy has been achieved clinically, chemoresistance, either primary or acquired, greatly hinders clinical application of anticancer drugs [3]. The cellular signaling balance between survival and death is one of the determining factors in cancer cells' response to anticancer drugs. Consequently, increased survival and/or suppressed apoptosis signaling underlie some of the mechanisms for chemoresistance [4, 5]. Therefore, tipping the cellular signaling balance between survival and death towards the side of death could improve anticancer chemotherapy [4].

Cisplatin kills cancer cells through the crosslinking of DNA, leading to replicative DNA damage, which in turn activates the intrinsic apoptosis pathway [6, 7]. As a main MAP kinase activated by extracellular stimuli and intracellular stresses, JNK is activated for apoptosis by cisplatin [6, 8]. The MAP3K-MAP2K-JNK kinase cascade [9], where MKK4 and MKK7 phosphorylate JNK for its activation [10, 11], is often the target for cell death signaling. Additionally, the activity of JNK is negatively regulated by a group of MAPK phosphatases of which MKP1 is the major JNK suppressor [12]. Interestingly, cisplatin induces MKP1 expression [8], which is assumed to be a cytoprotective response to cisplatin in cancer cells. More recently, MKP1 is implicated in resistance to cisplatin in breast cancer [13, 14]. Although numerous mechanisms such as drug efflux and detoxification, DNA repair activation, and apoptosis inhibition are implicated in cisplatin resistance [10, 11], retaining MKP1 expression and suppressing JNK activity may blunt cytotoxicity induced by cisplatin in cancer cells.

Receptor-interacting protein 1 (RIP1) is important for cell survival signaling [15-19]. However, recent studies have revealed a pro-death role for RIP1 under certain conditions [20-23]. Therefore, RIP1 stands at a unique position for the mediation of signals induced by different stimuli for either cell survival or death. Recently, an oncogenic role for RIP1was proposed in glioblastoma [24]. We found that RIP1 is overexpressed in human lung cancers and RIP1 promotes cigarette smoke carcinogen-induced human bronchial epithelial cell transformation, supporting a lung cancer promoting role for RIP1 [25]. In this report, we investigated the role of RIP1 in cancer cells' response to chemotherapy and provided evidence that RIP1 participates in chemoresistance to cisplatin. RIP1 suppresses JNK activation through releasing miR-940-mediated suppression of MKP1 expression, which in turn attenuates the anticancer activity of cisplatin; and targeting the RIP1/miR-940/MKP1 pathway may sensitize platinum-based anticancer therapy.

## RESULTS

### RIP1 knockdown potentiates cisplatin-induced cytotoxicity involving JNK activation

**Figure 1:**
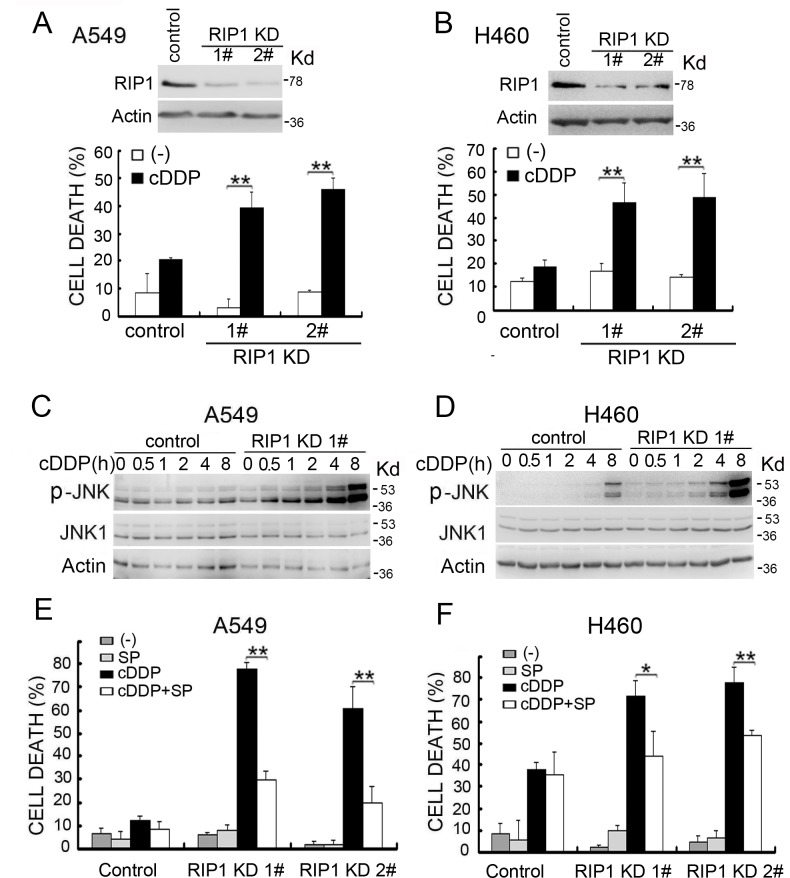
RIP1 knockdown potentiates cisplatin-induced cytotoxicity involving JNK activation (A, B) A549 and H460 cells (control and RIP1 stable knockdown clones 1 and 2) were treated with cisplatin (A549, 20 μM; H460, 10 μM) for 48 h. Cell death was detected with LDH release assay. Data shown are mean±SD. **p< 0.01. RIP1 knockdown was confirmed by Western blot, β-actin was used as an input control. (C, D) Cells were untreated or treated with cisplatin (A549, 20 μM; H460, 10 μM) for the indicated times. JNK and phospho-JNK were examined with Western blot. β-actin was detected as an input control. (E, F) Cells were pretreated with SP600125 (10 μM) for 30 min and then treated with cisplatin (A549, 20 μM; H460, 10 μM) for an additional 48 h, cell death was detected by LDH assay. Columns shown are mean±SD. *p<0.05,**p<0.01.

Stable RIP1 knockdown was established in A549 and H460 cells, dramatically increasing cisplatin-induced cytotoxicity (Fig. 1A, 1B). Because JNK is activated by cisplatin to induce apoptosis for killing cancer cells and RIP1 is involved in JNK induction by diverse stimuli [6, 8, 25-27], we examined cisplatin-induced JNK activation and found it was dramatically potentiated by RIP1 suppression (Fig. 1C, 1D). The specific pharmacological JNK inhibitor, SP600125, significantly lessened cisplatin-induced cell death (Fig. 1E, 1F). These results strongly suggest that RIP1 suppresses cisplatin-induced cytotoxicity through inhibiting JNK activation.

### JNK-dependent apoptosis induced by cisplatin in RIP1 knockdown cells

**Figure 2:**
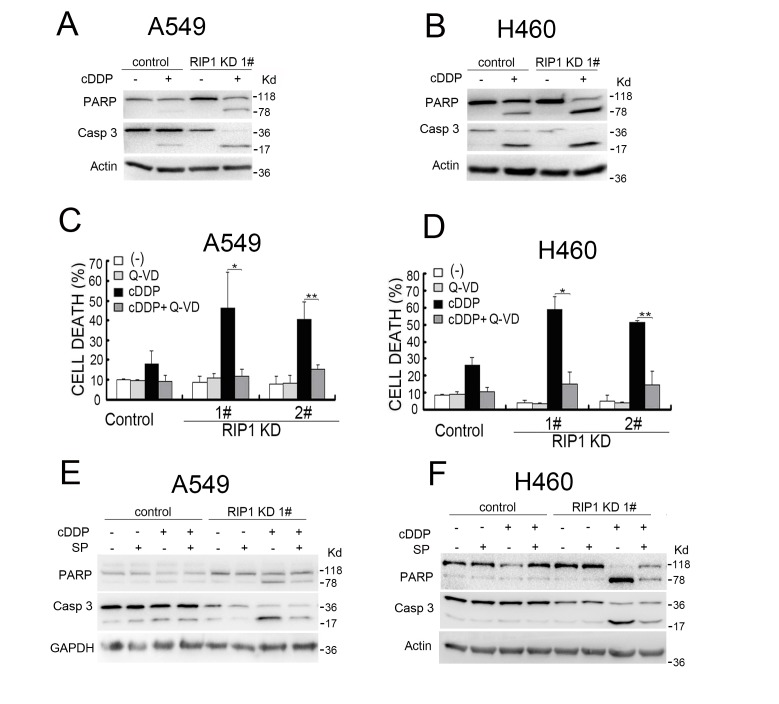
RIP1 knockdown potentiates cisplatin-induced and JNK-mediated apoptosis (A, B) A549 and H460 cells (control and RIP1 stable knockdown) were treated with cisplatin (A549, 20 μM; H460, 10 μM) for 24 h. Caspase 3 and PARP were detected with Western blot, β-actin was used as an input control. (C, D) Cells were pretreated with Q-VD (10 μM) for 30 min and then treated with cisplatin (A549, 20 μM; H460, 10 μM) for an additional 48 h, cell death was detected by LDH assay. Columns shown are mean±SD. *p<0.05,**p<0.01. (E, F) Cells were pretreated with SP600125 (10 μM) for 30 min and then treated with cisplatin (A549, 20 μM; H460, 10 μM) for an additional 24 h, Caspase 3 and PARP were detected with Western blot, β-actin was used as an input control.

Because cisplatin kills cancer cells through inducing apoptosis and JNK is activated in apoptosis [6-8], we examined if RIP1 regulates cisplatin-induced cytotoxicity through JNK-mediated apoptosis. Cisplatin-induced apoptosis, exhibited as enhanced activation of caspase 3 and cleavage of PARP, was enhanced in RIP1 knockdown cells (Fig. 2A, 2B). The pan-caspase inhibitor Q-VD and z-VAD attenuated cisplatin-induced cytotoxicity in RIP1 knockdown cells (Fig. 2C, 2D and data not shown). In addition, the JNK inhibitor, SP600125, significantly suppressed cisplatin-induced activation of caspase 3 and cleavage of PARP in RIP1 knockdown cells (Fig. 2E, 2F). These results suggest that RIP1 suppresses cisplatin-induced apoptosis by inhibiting JNK activation.

### Reduced MKP1 expression contributes to cisplatin-induced JNK activation and cytotoxicity in RIP1 knockdown cells

**Figure 3:**
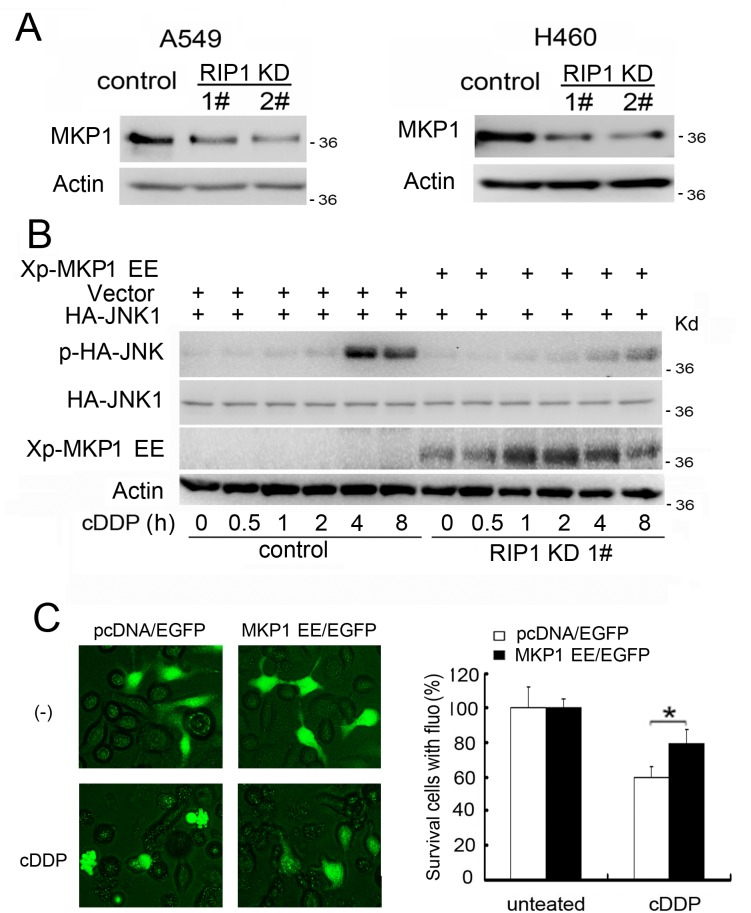
Reduced MKP1 expression contributes to cisplatin-induced JNK activation and cytotoxicity in RIP1 knockdown cells (A) MKP1 was detected with Western blot. β-actin was used as the input control. (B) pcDNA-MKP1 EE or the empty vector pcDNA were co-transfected with HA-JNK1 in A549 RIP1 knockdown cells (1#). The cells were treated with cisplatin (20 μM) for indicated times and JNK1, phospho-JNK1 and Xp-MKP1 EE were detected with Western blot with anti-HA, -phospho-JNK and –Xpress antibody, respectively. β-actin was used as an input control. (C) The cells were transfected with pcDNA-MKP1 EE or the empty vector pcDNA with EGFP in A549 RIP1 knockdown cells (1#) and treated with cisplatin (20 μM) for 24 h. Photographs were taken under a fluorescence microscope. Cell survival was quantified by counting cells with green fluorescence and normal morphology. Data shown was the mean ± SD, *p<0.05.

To elucidate the mechanism through which RIP1 contributes to cisplatin-induced JNK activation, we investigated if JNK inactivation is involved in RIP1-mediated JNK suppression by focusing on MKP1, a major JNK phosphatase involved in cisplatin resistance [13, 14]. The expression of MKP1 was significantly reduced in both RIP1 knockdown A549 and H460 cells (Fig. 3A). To determine if MKP1 reduction was the main reason for JNK activation, MKP1 EE, a degradation-resistant and enzymatically active MKP1 mutant [28], was used to reconstitute MKP1 activity in RIP1 knockdown cells. Ectopic expression of MKP1 EE dramatically suppressed cisplatin-induced JNK activation (Fig. 3B), and significantly rescued cells from cisplatin-induced cytotoxicity (Fig. 3C). These results suggest that MKP1 suppression plays a major role in cisplatin-induced JNK activation and cytotoxicity in RIP1 knockdown cells.

### Reduced MKP1 protein synthesis rate contributes to reduced MKP1 expression in RIP1 knockdown cells

**Figure 4:**
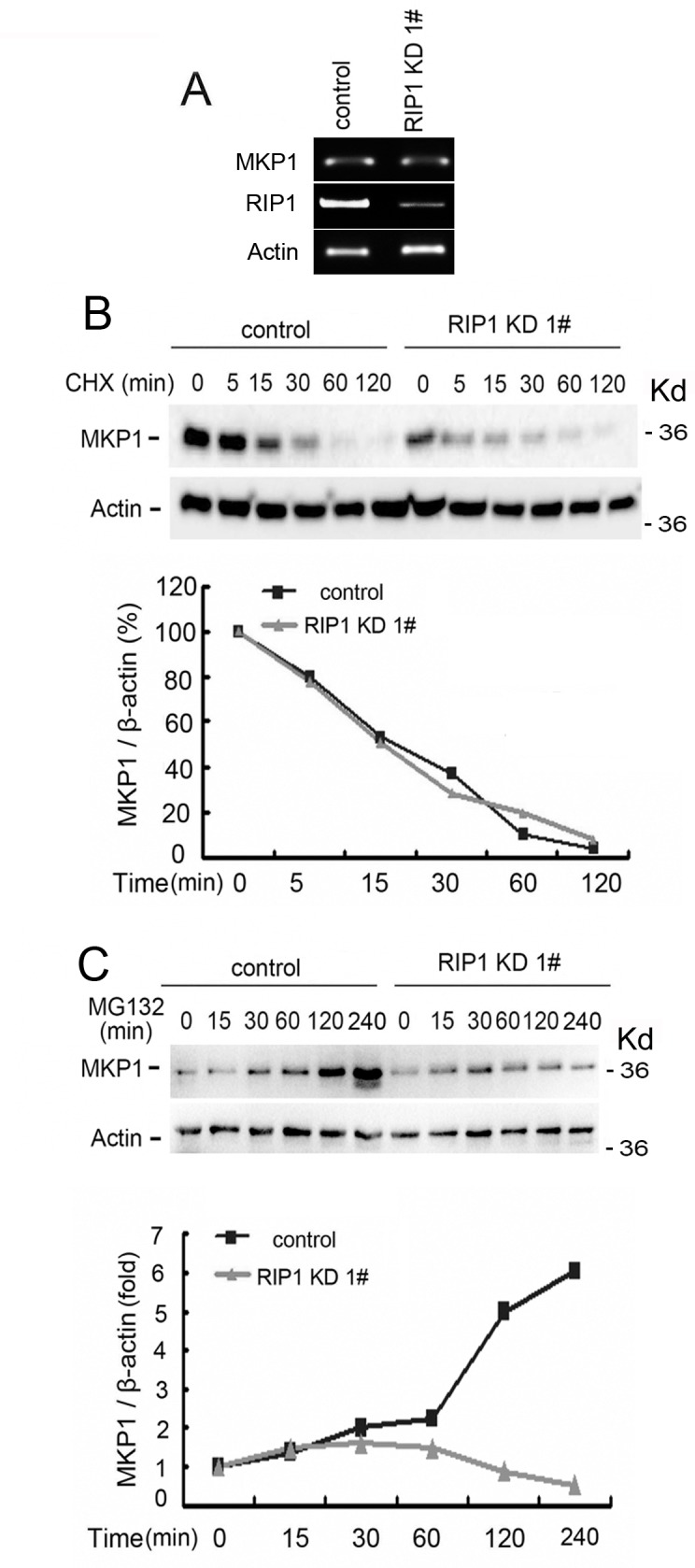
Reduced MKP1 protein synthesis rate in RIP1 knockdown cells (A) A549 cells (control and RIP1 knockdown) were collected for RNA isolation, MKP1 and RIP1 mRNA levels were detected using RT-PCR, β-actin was detected as an input control. (B) A549 cells (control and RIP1 knockdown) were treated with cycloheximide (CHX, 10 μg/ml) for the indicated time points. MKP1 was detected with Western blot. β-actin was used as the input control. The intensity of the individual bands was quantified by Quantity One® Software and normalized to the corresponding input control (β-actin) bands. (C) A549 (control and RIP1 knockdown) cells were pretreated with cycloheximide (CHX, 10 μg/ml) for 16 h to remove existing MKP1 protein, then the culture medium was refreshed and cells were treated with the proteasome inhibitor MG-132 (10 μM) for the indicated times. MKP1 and β-actin were detected with Western blot. The intensity of the individual bands was quantified as described in (B).

We then examined the mechanism through which MKP1 expression was reduced in RIP1 knockdown cells. The expression levels of MKP1 mRNA were hardly changed, while the protein concentrations were significantly lowered (Fig. 3A, 4A), suggesting a posttranscriptional mechanism for MKP1 reduction. In addition, when compared to control cells, the MKP1 protein degradation rate was unaltered in RIP1 knockdown cells (Fig. 4B). However, by examining the accumulation of newly synthesized protein through shut down of proteasomal protein degradation with MG132, the protein synthesis rate detected was significantly suppressed in RIP1 knockdown cells (Fig. 4C). These results strongly suggest that reduced MKP1 protein synthesis but not increased protein turnover contributes to suppressed MKP1 expression in RIP1 knockdown cells.

### Increased miR-940 is responsible for MKP1 protein expression suppression in RIP1 knockdown cells

**Figure 5:**
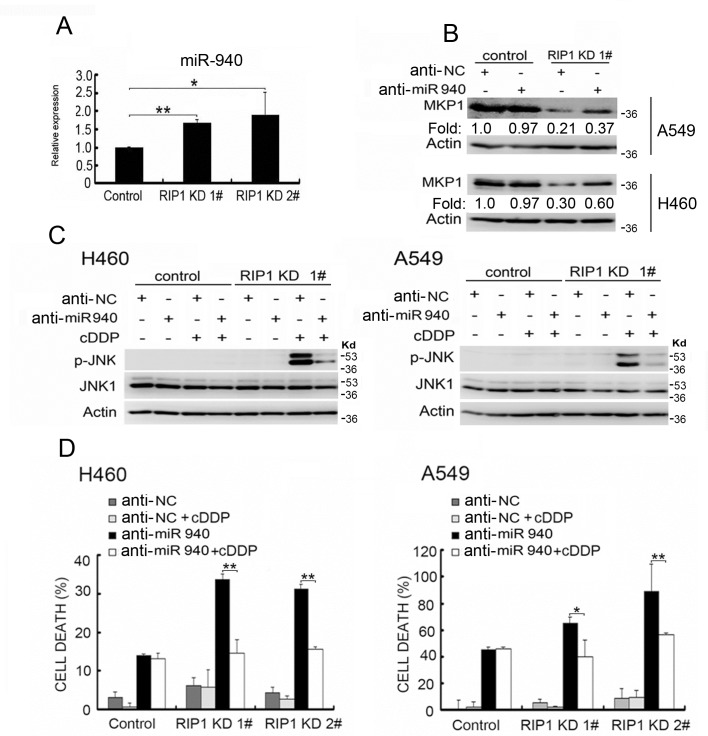
Increased miR-940 expression is involved in MKP1 suppression in RIP1 knockdown cells (A) Total RNA isolated from A549 cells (control and RIP1 knockdown) was used for detection of miR-940 with qPCR. (B) The cells were transfected with negative control or miR-940 miScript miRNA inhibitor for 48 h, MKP1 expression was detected with Western blot, and β-actin was detected as an input control. The intensity of the individual bands was quantified by Quantity One® Software and normalized to the corresponding input control (β-actin) bands. (C) The cells were transfected with the indicated miRNA inhibitor (10 nM) for 24h, and treated with cisplatin (20 μM) for 8 h. JNK1 and phospho-JNK1 were detected with Western blot. β-actin was detected as an input control. (D) The cells transfected with negative control or miR-940 inhibitor (10nM) for 24h, then the cells were left untreated or treated with cisplatin (20 μM) for an additional 48 h. Cell death was detected with LDH assay. Data shown are the mean±SD. *p<0.0, **p<0.01.

Recent studies have demonstrated that microRNA-mediated suppression of translation is a major mechanism for gene expression regulation [29]. Thus, we explored potential microRNA-mediated regulation of MKP1. In a search with miRWalk (http://www.umm.uni-heidelberg.de/apps/zmf/mirwalk/), miR-940 was suggested to be a potential MKP1 regulating microRNA. Using an miRNA microarray assay, miR-940 was found to be among the increased miRNAs in RIP1 knockdown A549 cells (data not shown). Using quantitative PCR, we confirmed that miR-940 was increased in RIP1 knockdown cells (Fig. 5A). Knockdown of miR-940 effectively restored MKP1 expression in RIP1 knockdown cells but had little effect on the MKP1 expression level in the control cells (Fig. 5B). Consistent with the JNK inactivating role of MKP1, knockdown of miR-940 to restore MKP1 expression strongly suppressed cisplatin-induced JNK activation (Fig. 5C). Furthermore, knockdown of miR-940 effectively suppressed cisplatin-induced cytotoxicity in RIP1 knockdown but not in the control cells (Fig. 5D). Taken together, these results suggest that RIP1 suppresses cisplatin-induced and JNK-mediated cytotoxicity through releasing the constraint of miR-940 on MKP1 expression.

### RIP1 knockdown potentiates cisplatin-induced MKK4 activation

**Figure 6:**
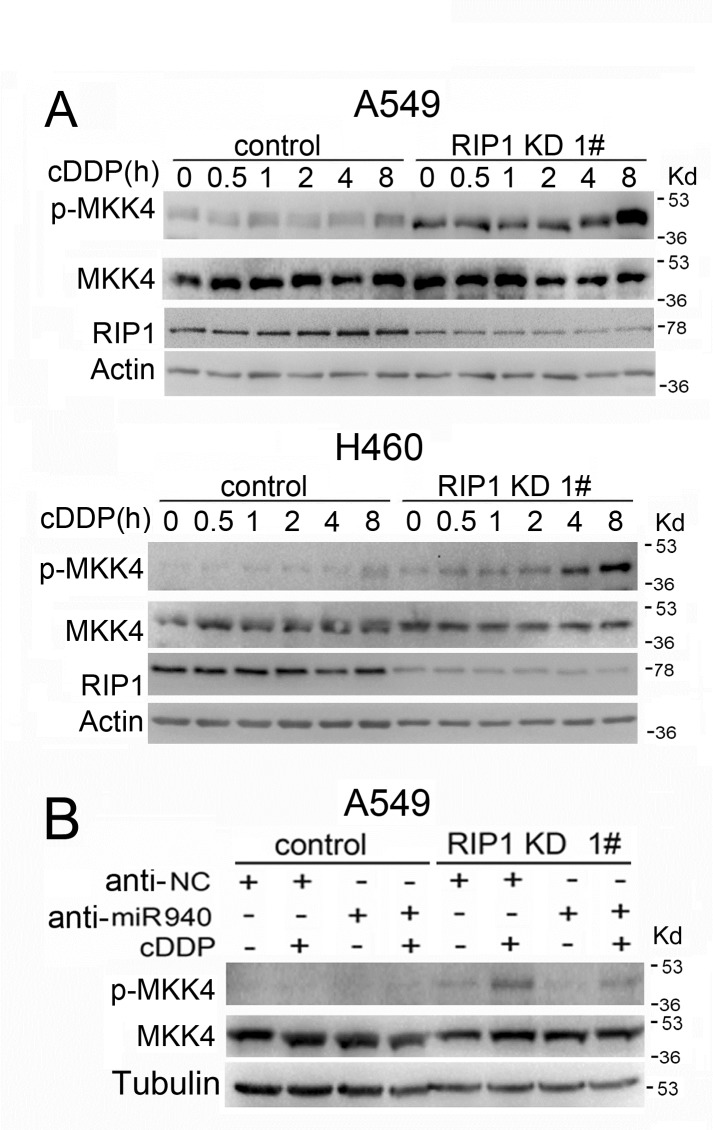
Cisplatin-induced MKK4 activation is enhanced in RIP1 knockdown cells (A). The cells were treated with cisplatin (20 μM for A549, 10 μM for H460) for indicated times or remained untreated. Phospho-MKK4 and total MKK4 were detected with Western blot. β-actin was used as an input control. (B). The cells were transfected with the indicated miRNA inhibitor (10 nM) for 24h, and treated with cisplatin (20 μM) for 8 h. Phospho-MKK4 and total MKK4 were detected by Western blot. β-tubulin was detected as an input control.

We also examined the upstream activating cascade for JNK. While no detectable activation of MAP3Ks, such as ASK or MEKK1, were detected in either control or RIP1 knockdown cells, a clear activation of MKK4 was detected in RIP1 knockdown cells (Fig. 6A, data not shown). Notably, the basal level of MKK4 activity in RIP1 knockdown A549 and H460 cells was elevated compared to the control cells (Fig. 6A). MKK7 activation, another MAP2K known for JNK activation, was not detected in either control or RIP1 knockdown cells (data not shown). These results suggest that RIP1 suppressed cisplatin-induced JNK activation also involves blocking of MKK4 activation.

We further examined whether miR-940 has a direct impact on MKK4 activation induced by cisplatin. The results show that knockdown of miR-940 had no detectable effect on MKK4 expression levels, suggesting that this microRNA does not regulate MKK4 expression. However, miR-940 knockdown slightly suppressed phospho-MKK4 in RIP1 suppressed cells (Fig. 6B), suggesting that miR-940 indirectly potentiates cisplatin-induced MKK4 activation when RIP1 is suppressed and these underlying mechanisms deserve further study.

## DISCUSSION

**Figure 7:**
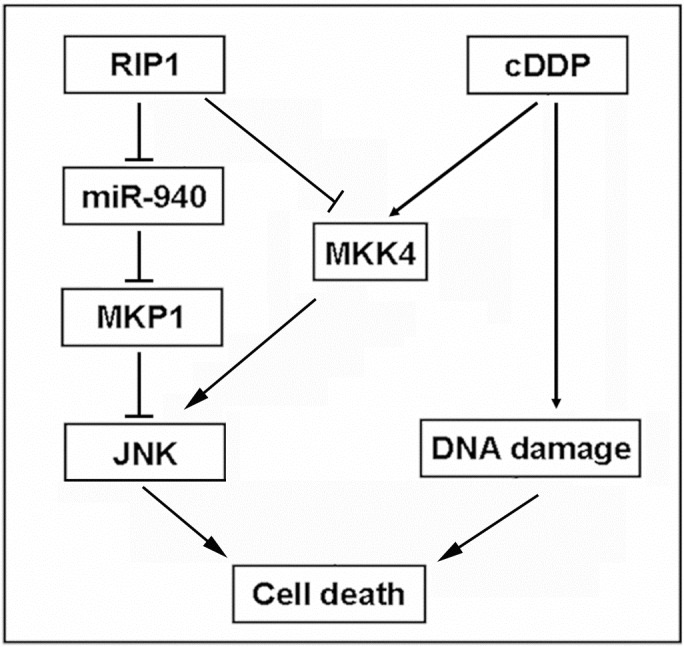
A model of RIP1 in regulation cisplatin-induced apoptosis RIP1 suppresses cisplatin-induced JNK activation through a dual mechanism: to release miR940-mediated suppression of MKP1 expression and to block MKK4 activation, which results in apoptosis suppression and cisplatin resistance.

In this report, we show evidence substantiating a cytoprotective role for RIP1 in lung cancer cell's response to cisplatin that includes suppression of JNK-mediated apoptotic cytotoxicity. RIP1 knockdown substantially increased cisplatin-induced apoptosis in lung cancer cells that was dependent on JNK activation. The synthesis rate of the JNK inactivating phosphatase, MKP1, was reduced in RIP1 knockdown cells. Furthermore, the expression of miR-940 was remarkably increased in RIP1 knockdown cells, and knockdown of this microRNA restored MKP1 expression and attenuated cisplatin-induced JNK activation and cytotoxicity. Importantly, ectopic MKP1 expression effectively attenuated cisplatin-induced JNK activation and cytotoxicity. We also show that RIP1 suppresses cisplatin-induced JNK activation entailing MKK4 blockage. Thus, our results suggest that RIP1 suppresses JNK activation through release of miR940-mediated suppression of MKP1 expression and blockage of MKK4, resulting in cisplatin resistance (Fig. 7). Interventions targeting this JNK activation pathway may sensitize platinum-based anticancer therapy.

While RIP1 was reported to mediate cytotoxicity for anticancer drugs under some circumstances [30-32], a cytoprotective role for RIP1 is documented [15, 17-19, 33]. The main mechanism for RIP1 involvement in resistance to cancer therapeutics is supposedly its mediation of cell survival signals such as NF-κB and Akt [15, 24, 34, 35]. Recently we found that RIP1 mediates autophagy to attenuate TRAIL's cytotoxicity in cancer cells [36]. In this report, we identify a novel cytoprotective mechanism for RIP1 that involves suppression of JNK-mediated apoptosis signaling. Thus, consistent with previous reports, these results suggest that the role of RIP1 in cancer cells' response to therapy is complex, and may depend on cell context or cytotoxicity inducers, and the cytoprotective function of RIP1 can be either due to survival promotion or suppression of apoptosis signaling.

While the MAP3K-MAP2K-JNK kinase cascade is a major target for JNK regulation due to many cellular stresses [9], cisplatin-induced activation of MKK4 was remarkably increased in RIP1-suppressed cells. Furthermore, our results indicate that JNK activation by cisplatin relies on reduced MKP1 expression in RIP1 knockdown cells. The critical role of MKP1 in suppressing cisplatin-induced JNK activation and cell death was substantiated with restoration of MKP1 activity by introduction of a constitutive active MKP1 mutant into the RIP1-suppressed cells. These results highlight MKP1 as an important factor for cancer cell survival and resistance to cisplatin, which is consistent with the reported role of MKP1 in breast and ovarian cancer cells [37-39].

Our results further suggest that the suppression of protein synthesis by miR-940 is the major mechanism for MKP1 expression reduction in RIP1 knockdown cells. MKP1 expression is controlled at multiple levels including that of transcription, translation and protein stability [13]. We previously found that the dietary flavonoid, luteolin, suppresses MKP1 expression through enhancement of proteasomal degradation in lung cancer cells [28]. Thus, various mechanisms are involved in regulation of MKP1 expression. Interestingly, we also found that in RIP1 knockdown lung cancer cells, miR-146a was increased, leading to catalase suppression [40]. Altogether, these results imply that RIP1 controls different microRNAs in order to control cancer cells' response to stresses. How RIP1 controls these microRNAs' expression is currently unclear. It would be interesting to determine if RIP1-mediated pathways are involved in controlling the expression of miR-940 or if RIP1 acts as a co-activator for miR-940 transcription. The latter possibility was supported by the fact that RIP1 localizes to the nucleus and RIP1 also regulates transcription of some protein-coding genes [15, 41].

It is of note that while apoptosis induction underlies one of the major functions of chemotherapeutics, recent studies highlight that necrotic cell death is also involved in chemotherapeutic-induced cancer cell death. Some forms of necrotic cell death in cancer cells depend on formation of the RIP1-containing complex Ripoptosome, while other subgroups of necrosis may not involve RIP1 [23, 42, 43]. In addition, there is crosstalk between the apoptosis and necrosis signaling pathways and the modes of cell death are cell context- and stimulation-specific [44]. Therefore, more in depth mechanistic studies are necessary to investigate the role of RIP1 in cancer cell necrosis, which will likely lead to potential new adjuvant cancer therapies for improving chemotherapy efficacy.

In summary, we identify a dual mechanism for suppressing cisplatin-induced and JNK-mediated cytotoxicity by RIP1 through releasing miR-940-mediated MKP1 suppression and inhibiting MKK4 activation. While further mechanistic studies are warranted, interventions targeting this RIP1-mediated cytoprotective signaling pathway may sensitize platinum-based chemotherapy.

## MATERIALS AND METHODS

### Reagents

Cisplatin (479306) was from Sigma (St. Louis, MO). RIP1 (610458) and JNK1 (544286) antibodies were from BD Biosciences (San Jose, CA). MKP1 (sc-370), HA (sc-805), -Xpress (sc-499) and GAPDH (sc-32233) antibodies were from Santa Cruz Biotechnology (Dallas, TX), and β-actin (A1978) was from Sigma (St. Louis, MO). Phospho-JNK (44-682G) antibody was from Invitrogen (Camarillo, CA). Anti-phospho-MKK4 (cs-9156s) and total MKK4 (CS-9152) were from Cell Signaling Technology (Danvers, MA). MG-132 (474790) was from Calbiochem. Chloroquine (C6628) and Cycloheximide (C1988) were from Sigma. Anti-hsa-miRNA-940 (MIN0004983) miScript miRNA inhibitor targeting miR-940 was from QIAGEN (Germantown, MD).

### Cell Culture

Non-small cell lung cancer cell lines A549 and H460 were obtained from American Type Culture Collection (Manassas, VA) and grown in RPMI 1640 supplemented with 10% fetal bovine serum, 2mM L-glutamine, 100 units/mL penicillin, and 100 μg/mL streptomycin. All cells were grown under standard incubator conditions at 37°C, with 5% CO_2_.

### Lentivirus infection and establishment of stable cell lines

Lentivirus vectors with short hairpin RNAs (shRNA) against RIP1 and control vectors were purchased from Open Biosystems (Lafayette, CO). Viruses were produced and packaged in HEK293T cells following to manufacturer's instruction. A pLKO.1 backbone harboring the shRNA sequence CCGGAGGTCATGTTCTTTCAGCTTACTCGAGTAA GCTGAAAGAACATGACCTTTTTT (mature sequence: AGGTCATGTTCTTTCAGCTTA; Cat. No. RHS3979-9569092) was used to establish A549 RIP1 knockdown cell lines. H460 RIP1 knockdown cell line were created with the pGIPZ vector and the shRNA sequence TGCTGTTGACAGTGAGCGCGCAGTTGATAATGTG CATAAATAGTGAAGCCACAGATGTATTTATG CACATTATCAACTGCTTGCCTACTGCCTCGGA (mature sequence: TTATGCACATTATCAACTG; Cat. No. RHS4430-98902904). Cells were infected with viruses and selected with 5 μg/ml of puromycin. Positive clones were expanded and maintained in medium supplemented with 1 μg/ml of puromycin.

### Western Blot

Cells were lysed in M2 buffer (20 mM Tris-HCl pH 7.6, 0.5% NP40, 250 mM NaCl, 3 mM EDTA, 3 mM EGTA, 2 mM DTT, 0.5 mM phenylmethylsulfonyl fluoride, 20 mM β-glycerophosphate, 1 mM sodium vanadate, and 1 μg/mL leupeptin). Cell lysates with equal amounts of protein were resolved on 12% or 15% SDS-PAGE and transferred to a polyvinylidene fluoride membrane, then detected with various antibodies. The proteins were visualized with enhanced chemiluminescence (Millipore), following manufacturer's instructions.

### Cytotoxicity assay

Cell death was assessed based on the release of lactate dehydrogenase (LDH) with a cytotoxicity detection kit (Promega) using a previously described protocol [45]. Cells were seeded in 48-well plate one day before treatment and then treated as indicated in each figure legend. Quantification of cell death was as previously described [46].

### microRNA expression analysis

Total RNA was isolated from cells with TRIzol Reagent (Life Technologies, Grand Island, NY). Total RNA (1μg) was reverse transcribed with the miScript II RT Kit (QIAGEN). Quantitative real-time PCR was carried out with the ABI PRISM 7900HT using Power SYBR Green PCR Master Mix (Applied Biosystems). Experiments were normalized to RNU6b. Data were analyzed as RQ with respect to a calibrator sample using the 2^−ΔΔCT^ method [45].

### Fluorescence microscopy

RIP1 knockdown A549 cells were transfected with EGFP and pcDNA or EGFP and pcDNA-MKP1 EE expression plasmids, and then treated with cisplatin (20 μM) for 24 h and examined under a fluorescence microscope. Images shown are representative of three experiments. The percentage of fluorescent cells was calculated.

### Statistics

All data were expressed as means ±SD and examined by Student's *t*-test for statistical significance. p<0.05 was considered statistically significant.
